# Pharmacokinetic and Pharmacodynamic Target Attainment in Adult and Pediatric Patients Following Administration of Ceftaroline Fosamil as a 5‐Minute Infusion

**DOI:** 10.1002/cpdd.907

**Published:** 2021-01-19

**Authors:** Todd A. Riccobene, Timothy J. Carrothers, William Knebel, Susan Raber, Phylinda L.S. Chan

**Affiliations:** ^1^ AbbVie Madison New Jersey USA; ^2^ Metrum Research Group Inc Tariffville Connecticut USA; ^3^ Pfizer, La Jolla California USA; ^4^ Pfizer, Sandwich Kent UK

**Keywords:** ceftaroline fosamil, infusion duration, population pharmacokinetics, PK/PD, probability of target attainment

## Abstract

The key pharmacokinetic/pharmacodynamic (PK/PD) efficacy index for β‐lactam antibiotics is the percentage of time that free drug concentrations exceed the minimum inhibitory concentration (MIC) of bacteria during each dosing interval (*f*T>MIC). Ceftaroline fosamil, the prodrug of the β‐lactam ceftaroline, was initially approved for administration as 60‐minute intravenous (IV) infusions. Population PK analyses comparing exposure and PK/PD target attainment for 5‐minute and 60‐minute IV infusions, described here, have supported ceftaroline fosamil labeling updates to include variable infusion durations of 5 to 60 minutes in adults and children aged ≥2 months. A 2‐compartment disposition PK model for ceftaroline fosamil and ceftaroline was used to predict steady‐state ceftaroline exposures (maximum plasma concentrations [C_max,ss_] and area under the plasma concentration–time curve over 24 hours [AUC_ss,0‐24_]) and probability of target attainment in simulated adult and pediatric patients with various degrees of renal function receiving standard doses of ceftaroline fosamil as 5‐minute or 60‐minute IV infusions. Across age groups and renal function categories, median ceftaroline AUC_ss,0‐24_ values were similar for 5‐minute and 60‐minute infusions, whereas C_max,ss_ was up to 42% higher for 5‐minute infusions. Both infusion durations achieved >99% probability of target attainment based on PK/PD targets for *Staphylococcus aureus* (35% *f*T>MIC) and *Streptococcus pneumoniae* (44% *f*T>MIC) at European Committee on Antimicrobial Susceptibility Testing/Clinical and Laboratory Standards Institute MIC breakpoints (1 mg/L and 0.25/0.5 mg/L, respectively). These findings support administration of standard ceftaroline fosamil doses over 5 to 60 minutes for adults and children aged ≥2 months, providing added flexibility to clinicians and patients.

Ceftaroline, the active metabolite of the prodrug ceftaroline fosamil, is a β‐lactam antibiotic with in vitro activity against Gram‐positive bacteria, including *Staphylococcus aureus* (methicillin‐susceptible and ‐resistant strains) and *Streptococcus pneumoniae*, and common Gram‐negative pathogens that do not express extended‐spectrum β‐lactamase enzymes.^1^, ^2^, ^3^ Ceftaroline fosamil is rapidly converted to active ceftaroline by plasma phosphatases upon intravenous (IV) administration. Ceftaroline exhibits linear pharmacokinetics (PK) with plasma clearance in healthy subjects of 10 L/h, renal clearance of 4 to 7 L/h, volume of distribution of 30 to 40 L, and half‐life of ≈2.6 hours.^3^, ^4^, ^5^ Elimination is via the renal route, with dose adjustments required for patients with moderate or severe renal impairment.^6^, ^7^, ^8^ In population PK models, renal function, age, and presence of infection had significant effects on ceftaroline clearance; ceftaroline clearance is unaffected by race/ethnicity or sex.^7^, ^8^

For β‐lactam antibiotics, including ceftaroline, the percentage of a dosing interval that free drug concentrations exceed the minimum inhibitory concentration (MIC) of target bacteria (%*f*T>MIC) is considered the pharmacodynamic (PD) index most closely associated with clinical efficacy.^9^, ^10^ Hence, to optimize probability of target attainment (PTA), frequent dosing with prolonged infusions or continuous infusions have been recommended for severely ill patients undergoing β‐lactam treatment.^11^, ^12^, ^13^

For ceftaroline fosamil, standard doses are given every 8 or 12 hours, with product labeling originally specifying a 1‐hour infusion duration. Population PK modeling and Monte Carlo simulations based on patient PK data from the adult and pediatric ceftaroline fosamil clinical development programs have shown that standard ceftaroline fosamil doses administered by 1‐hour infusion are expected to achieve >90% PTA in adults and pediatric patients aged ≥2 months against target pathogens including *S. aureus* and *S. pneumoniae* based on pathogen‐specific *f*T>MIC targets.^6^, ^7^, ^8^ Population PK modeling and Monte Carlo simulations have also supported the use of ceftaroline fosamil (standard doses) in neonates and young infants <2 months in Europe and the United States,^14^ and high‐dose recommendations for treatment of adults and children aged ≥2 months with complicated skin and soft skin infections (cSSTI) caused by rare *S. aureus* isolates with ceftaroline MICs of 2 to 4 mg/L in Europe.^8^, ^15^

Shorter IV infusions of antibiotics can provide clinical and practical advantages over longer infusions, for example, in the emergency department, in fluid‐restricted patients, in ambulatory patients requiring IV treatment, and in the context of intravenous fluid and/or fluid bag supply shortages.^16^, ^17^, ^18^, ^19^ Based on well‐established principles, for a given dose of a drug with linear PK, the impact of a varying IV infusion duration would be on the magnitude of maximum plasma concentration (C_max_) and shape of the concentration–time curve during administration and the distribution phase; net area under the plasma concentration–time curve (AUC) and mean steady‐state concentration (Css) during the dosing interval would remain unchanged.^20^ For shorter infusions of β‐lactams, an important consideration is whether the shape of the concentration–time profile (arising from the higher C_max_ and earlier time to maximum concentration) is shifted such that *f*T>MIC is changed to an extent that affects PK/PD target attainment. Moreover, the potential for adverse effects arising from more rapid infusions and higher C_max_ values should also be considered.

This report summarizes the results of the modeling and simulations to support the use of a variable IV infusion duration of 5–60 minutes for standard ceftaroline fosamil doses in patients aged ≥2 months (approval in the United States in 2015 and in Europe in 2019).

## Methods

### Population PK Models

Predictions of ceftaroline steady‐state exposures and PTA simulations for pediatric patients and adults with normal renal function were performed using a previously reported population PK model for ceftaroline fosamil and ceftaroline.^6^ The model included data from 525 adult and pediatric patients with community‐acquired pneumonia or cSSTI and 195 healthy subjects, contributing 6633 measurable concentrations (1799 for ceftaroline fosamil and 4834 for ceftaroline).^6^ Concentrations were measured using validated liquid chromatography–tandem mass spectrometry bioanalytical methods.^7^, ^21^, ^22^ Samples with concentrations below the lower limit of quantification were excluded from the analyses.^6^ To compare exposures and PTA in renally impaired pediatric patients with those in adults with mild, moderate, or severe renal impairment, a separate, previously unreported population PK model comprising data from 227 adult patients and 219 healthy subjects was used for simulations of adults with renal impairment. The data set for the adult renal impairment model included 17 phase 1 to 3 clinical trials, including a single‐dose study of 5‐ and 60‐minute IV infusions in healthy subjects. Further details of the population PK models and analyses are provided in the Supplemental Methods.

### Pharmacokinetic/Pharmacodynamic Targets

PK/PD targets for ceftaroline against *S. aureus* were determined from preclinical models including a neutropenic murine thigh infection model, an in vitro single‐compartment dilutional PK model, and an in vitro hollow fiber infection model.^8^, ^9^, ^23^, ^24^ Cumulatively, 24 molecularly diverse *S. aureus* isolates with ceftaroline MICs ranging from 0.12 to 4 mg/L were studied. Mean *f*T>MIC values derived from these studies were 27% for bacterial stasis, 31% for 1‐log_10_ colony‐forming units/mL reduction in bacterial density, and 35% for 2‐log_10_ reduction. A ceftaroline PK/PD target of 44% *f*T>MIC for *S. pneumoniae* (associated with 1‐log_10_ bacterial killing) was derived from studies using a neutropenic murine thigh and lung infection model, involving *S. pneumoniae* isolates with ceftaroline MICs of 0.008 to 0.12 mg/L.^9^

### Monte Carlo Simulations

Monte Carlo simulations using the final population PK models, appropriate covariate distributions, and a predefined level of parameter uncertainty, were performed to predict ceftaroline C_max,ss_ and AUC_ss,0‐24_ and to calculate PTA by MIC based on the above PK/PD targets for various ceftaroline fosamil dosage regimens (5‐minute and 60‐minute IV infusions), age, and renal function groups. Comparisons between the 5‐minute and 60‐minute infusion durations were based on ceftaroline plasma exposures at steady state and achievement of 35% *f*T>1 mg/L for *S. aureus* and 44% *f*T>0.5 mg/L for *S. pneumoniae*, respectively. For *S. aureus*, the PK/PD target corresponds to the current European Committee on Antimicrobial Susceptibility Testing (EUCAST) and Clinical and Laboratory Standards Institute (CLSI) MIC susceptible breakpoint for standard‐dose ceftaroline fosamil.^25^, ^26^ For *S. pneumoniae*, the EUCAST ceftaroline susceptible breakpoint (MIC ≤0.25 mg/L) differs from that of CLSI (MIC ≤0.5 mg/L); the current analysis used the higher CLSI breakpoint.^25^, ^26^ For pediatric patients, 100 simulations were performed for each dosage regimen and renal function category, with 600 (300 male and 300 female) patients in each 1‐month age group from ≥2 months to <18 years (60 000 simulated patients total per age group/regimen). For adults, 300 patients were simulated for each dosage regimen and renal function category for each of the 100 simulated data sets (30 000 simulated patients total per renal function group/regimen). Target attainment simulations were adjusted for plasma protein binding of ceftaroline, assumed to be 20%.

## Results

### Predicted Ceftaroline Exposures

**Figure 1:**
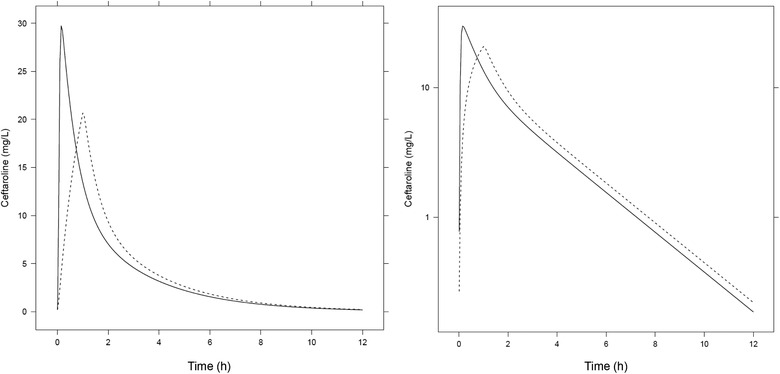
Model‐predicted ceftaroline concentration–time profile at steady state for a typical adult patient with normal renal function (nCrCL ≥80 mL/min/1.73 m^2^) receiving ceftaroline fosamil 600 mg every 12 hours as 5‐minute (solid lines) and 60‐minute (dashed lines) intravenous infusions. Y‐axis in linear (left panel) and logarithmic (right panel) scale. Typical adult patient with body weight 70 kg. nCrCL, body surface area‐normalized creatinine clearance.

Model‐predicted median steady‐state ceftaroline exposures in pediatric and adult patients with normal renal function are shown in Table 1, and plasma concentration–time profiles for a typical adult with normal renal function receiving 5‐minutes and 60‐minutes infusions are shown in Figure 1. Predicted ceftaroline exposures in adults and pediatric patients with mild, moderate, or severe renal impairment are provided in Tables S1 through S3, respectively.

**Table 1:** Model‐predicted Median (90% Prediction Interval) Steady‐State Ceftaroline Exposure Parameters for Simulated Patients With Normal Renal Function (nCrCL ≥80 mL/min/1.73 m^2^) Receiving Ceftaroline Fosamil as 5‐Minute and 60‐Minute IV Infusions

Age Group	Dosage Regimen^a^	IV Infusion Duration	Weight (kg)^b^	C_max,ss_ (mg/L)^b^	C_max,ss_ Ratio^c^	AUC_ss,0–24_ (mg • h /L)^b^	AUC_ss,0–24_ Ratio^c^	%*f*T>1 mg/L^b^
Adults	600 mg every 12 h	60 min	77.6 (52.2‐105)	20.8 (11.7‐36.4)	1.27	97.5 (59.1‐164)	1.00	64.5 (45.0‐93.4)
		5 min		26.5 (13.8‐52.0)		97.2 (58.6‐164)		60.3 (41.3‐90.1)
>12 to <18 y	12 mg/kg every 8 h	60 min	52.9 (36.8‐75.3)	19.7 (11.0‐34.2)	1.30	122 (72.7‐201)	1.00	84.0 (59.3‐100)
		5 min		25.7 (13.1‐50.7)		122 (78.3‐203)		79.0 (53.1‐100)
≥6 to <12 y	12 mg/kg every 8 h	60 min	28.5 (19.3‐46.5)	27.6 (16.4‐43.3)	1.36	157 (99.7‐245)	1.00	85.2 (61.7‐100)
		5 min		37.4 (20.0‐69.2)		157 (99.7‐247)		79.0 (55.6‐100)
≥2 to <6 y	12 mg/kg every 8 h	60 min	15.8 (11.8‐22.2)	27.1 (16.8‐41.8)	1.42	144 (92.6‐225)	1.00	75.3 (54.3‐98.8)
		5 min		38.4 (21.2‐68.7)		144 (92.2‐222)		70.4 (49.3‐97.5)
18 to <24 mo	8 mg/kg every 8 h	60 min	11.7 (9.81‐14.1)	18.8 (11.8‐29.1)	1.39	107 (69.0‐165)	1.00	71.9 (51.9‐97.5)
		5 min		26.3 (14.7‐46.8)		107 (69.2‐166)		66.7 (46.9‐93.8)
12 to <18 mo	8 mg/kg every 8 h	60 min	10.4 (8.60‐12.7)	19.1 (11.9‐29.4)	1.38	113 (71.8‐174)	0.99	75.3 (54.3‐98.8)
		5 min		26.4 (14.9, 47.5)		112 (71.9, 174)		69.1 (48.1, 96.3)
6 to <12 mo	8 mg/kg q8h	60 min	8.43 (6.55‐10.7)	19.6 (12.2‐30.0)	1.36	120 (78.3‐188)	1.01	80.2 (58.0‐100)
		5 min		26.6 (14.9‐46.9)		121 (78.1‐188)		75.3 (53.0‐98.8)
2 to <6 mo	8 mg/kg every 8 h	60 min	5.75 (4.12‐7.66)	19.2 (12.1‐29.7)	1.31	134 (86.6‐208)	1.00	92.6 (66.7‐100)
		5 min		25.1 (14.4‐44.0)		134 (86.5‐209)		87.7 (60.5‐100)

%*f*T>MIC, percentage of time that free drug concentrations are above the minimum inhibitory concentration (MIC) of the bacteria during a dosing interval; AUC_ss,0‐24_, area under the plasma concentration–time curve over 24 hours at steady state; C_max,ss_, maximum plasma concentration for a dosing interval at steady state; IV, intravenous; nCrCL, body surface area‐normalized creatinine clearance.

^a^ All every 8 hours pediatric dosage regimens were up to a maximum of 400 mg based on weight.

^b^ Values are median and 5^th^ and 95^th^ percentiles (corresponding to 90% prediction intervals) based on summary of 100 simulation trials.

^c^ Ratios are for 5‐minute to 60‐minute IV infusions.

Predicted ceftaroline AUC_ss,0–24_ values were similar for the 5‐minute and 60‐minute infusions across all age groups and renal function categories. Given that within these groups, the same doses were given and only the duration of infusion varied, the AUC_ss,0‐24_ is expected to be the same or similar for 5‐minute and 60‐minute infusions. For adult and pediatric patients (aged ≥2 months to <18 years) with normal renal function, predicted C_max,ss_ values were 27% and 30% to 42% higher for the 5‐minute vs 60‐minute infusions (Table 1). Corresponding relative C_max,ss_ increases for patients with mild, moderate, or severe renal impairment were 18% to 26% and 17% to 39% (Tables S1–S3). The highest predicted median C_max,ss_ value across all age, dose, and renal function groups was in pediatric patients aged ≥2 to <6 years with mild renal impairment (39.3 mg/L, Table S2).

In adults with normal renal function, predicted median C_max,ss_ values were 20.8 mg/L (60‐minute infusion) and 26.5 mg/L (5‐minute infusion), a ratio of 1.27. Across all pediatric age groups and renal function categories, predicted median C_max,ss_ ratios did not exceed 1.42.

### Probability of Target Attainment Simulations

Results of the simulations estimating PTA for PK/PD targets of 35% *f*T>MIC of 1 mg/L for *S. aureus* and 44% *f*T>MIC of 0.5 mg/L for *S. pneumoniae* are shown in Table S4. Ceftaroline fosamil 5‐minute and 60‐minute IV infusions achieved similar PTA (≥99%) against *S. aureus* and *S. pneumoniae* at the respective PK/PD targets in all simulated age groups and renal function categories.

## Discussion

β‐Lactams, such as ceftaroline fosamil, demonstrate time‐dependent PD properties, with *f*T>MIC of the target organism being the PK/PD index most closely associated with antimicrobial efficacy.^27^, ^28^ For time‐dependent antibiotics, the duration of infusion may affect *f*T>MIC, and thus the probability of PK/PD target attainment could theoretically be impacted.^27^, ^29^ Population PK modeling and Monte Carlo simulations of exposure and PTA have extensive applications in antimicrobial drug development.^30^, ^31^, ^32^ For ceftaroline fosamil, modeling and simulations have supported adult and pediatric dose selection, dose adjustments for renal impairment, and determination of susceptibility breakpoints.^6^, ^7^, ^8^, ^33^ Our PK/PD analysis of a reduced IV infusion duration for standard doses of ceftaroline fosamil demonstrated negligible impact on PTA against 2 key target pathogens at their respective MIC susceptibility breakpoints.

Reducing infusion duration has also been evaluated for other time‐dependent antibiotics. A study evaluating the effect of a 5‐minute IV infusion duration vs standard 30‐minute infusions on the exposures of approved doses of meropenem, cefepime, and aztreonam demonstrated negligible effects on PTA.^34^ However, ceftaroline is one of a few antibiotics to have been so extensively evaluated from a clinical pharmacology perspective using modern modeling and simulation techniques. Health authority approval of 5‐ to 60‐minute variable IV infusions for standard ceftaroline fosamil doses in part reflects the extensive PK data accrued during clinical development and the robust iterative population PK modeling and simulations undertaken, both in adults and more recently in children.

The current analysis, comparing exposures and PK/PD target attainment for ceftaroline fosamil administered as 5‐minute and 60‐minute IV infusions in adults and pediatric patients, used population PK modeling and exposure simulations based on an extensive adult and pediatric PK data set.^6^, ^8^ The simulation results suggest that the standard ceftaroline fosamil dosage regimens achieved predicted plasma ceftaroline exposures associated with clinical efficacy in adults and pediatric patients when administered as 5‐minute or 60‐minute IV infusions.

In previous population PK analyses reported by Riccobene et al,^6^ which supported the initial pediatric approvals for standard ceftaroline fosamil doses, PK/PD targets of 36% and 44% for 1‐log_10_ reduction in bacterial density of *S. aureus* and *S. pneumoniae*, respectively, were used to determine PTA. The PK/PD target of 36% for 1‐log_10_ reduction for *S. aureus* was derived from a single neutropenic murine thigh infection study using *S. aureus* isolates with ceftaroline MICs of 0.12 to 1 mg/L.^6^, ^9^ A subsequent analysis by Das et al^8^ included additional studies carried out by MacGowan et al^23^ and Singh et al^24^ using an in vitro single‐compartment dilutional PK model and an in vitro hollow‐fiber model, respectively, to derive ceftaroline *S. aureus* PK/PD targets of 27% for stasis, 31% for 1‐log_10_ cfu/mL reduction in bacterial density, and 35% for 2‐log_10_ reduction. Collectively, these 3 studies included *S. aureus* isolates with a wider range of ceftaroline MICs (0.12‐4 mg/L) and genotypes relative to the single in vivo study alone.^8^, ^9^, ^23^, ^24^ Hence, 35% *f*T>MIC can be considered a robust PK/PD target.

Our PTA analysis for 5‐minute and 60‐minute IV infusions used the most recently characterized PK/PD targets^8^ at MIC values corresponding to EUCAST/CLSI MIC susceptible breakpoints for *S. aureus* (≤1 mg/L) and *S. pneumoniae* (≤0.25 and 0.5 mg/L, respectively).^25^, ^26^ At these MIC values, simulated adult and pediatric patients with mild, moderate, and severe renal impairment receiving standard ceftaroline fosamil doses as 5‐minute IV infusions achieved very high PTA (≥99%); although median C_max,ss_ values were up to 42% higher for 5‐minute infusions, the similar AUC_ss,0–24_ values ensured exposures were adequate to maintain high PTA. Interpretative criteria for antimicrobial susceptibility (breakpoints) such as those published by CLSI and EUCAST are typically derived from a combination of data including MIC distributions of target pathogens (wild‐type and resistant strains); molecular characterization of resistance mechanisms; PK/PD targets from animal models and in vitro studies; and patient data including PK exposures and clinical outcomes.^35^ As such, breakpoints are regularly reviewed and can be updated to reflect new data, or changes in dosing or administration regimens. The CLSI and EUCAST breakpoints used in the current analysis are based on extensive microbiological, PK/PD, and clinical data^6^, ^7^, ^8^, ^9^, ^23^, ^24^; nonetheless, as with all antimicrobial therapies, ceftaroline fosamil should only be used in line with approved labeling and for pathogen(s) known or suspected to be susceptible based on contemporary breakpoints.

Extensive reviews of ceftaroline PK, PD, and clinical outcomes have been previously reported.^3^, ^4^ In general, ceftaroline fosamil exhibits a favorable safety profile and is well tolerated.^36^ There were no major safety concerns reported in the phase 3 ceftaroline clinical trials, including in the high‐dose study in adults with cSSTI (600 mg every 8 hours by 2‐hour IV infusions) where the total daily dose (1800 mg) was 50% higher than the standard total daily dose (1200 mg).^36^, ^37^ For simulated adults with normal renal function, the magnitude of the elevated ceftaroline C_max_ for ceftaroline fosamil standard doses given as 5‐minute IV infusions (median, 26.5 mg/L vs 20.8 mg/L for 60‐minute infusions) is substantially lower than the mean ceftaroline C_max_ (81.4 mg/L) in a phase 1 single‐dose QT study in which healthy adults received ceftaroline fosamil 1500 mg 60‐minute IV infusions.^22^

For children, as the highest predicted median C_max,ss_ values in our analysis are comparable to the highest observed ceftaroline concentrations from sparse sampling in (1) a phase 2/3 study investigating the safety, tolerability, efficacy, and PK of standard‐dose ceftaroline in pediatric patients with acute bacterial skin and skin structure infections^38^; (2) a phase 4, high‐dose ceftaroline fosamil study in pediatric patients with complicated community‐acquired pneumonia^39^; and (3) a phase 4 single‐dose study evaluating PK, safety, and tolerability of ceftaroline fosamil in children (aged 0 to ≤12 years) with suspected or confirmed infection,^6^ in which no serious adverse events were related to high exposure (10 mg/kg up to a maximum of 600 mg, 60‐minute IV infusions), it is plausible to expect that safety would be maintained with a 5‐ to 60‐minute variable infusion duration. While faster infusions may theoretically increase local tolerability adverse events (eg, phlebitis), there was no difference in local tolerability adverse events in a phase 1 study of standard ceftaroline fosamil doses (60‐minute IV infusions) administered in reduced infusion volumes in adults.^17^

In conclusion, these data provide assurance that standard ceftaroline fosamil doses can be administered over a variable infusion duration of 5 to 60 minutes in patients aged ≥2 months without compromising exposure and PK/PD target attainment for *S. aureus* and *S. pneumoniae* with MICs within current EUCAST and CLSI susceptible breakpoints. Ongoing real‐world experience will help to establish the patient populations and treatment settings in which ceftaroline fosamil administered by shorter IV infusions might provide clinical and economic advantages.

## Conflicts of Interest

P.L.S.C. and S.R. are employees of and shareholders in Pfizer. T.R. and T.J.C. are employees of AbbVie (following its acquisition of Allergan). W.K. is president of Metrum Research Group Inc, which received funding from Allergan for support and assistance with the population PK analyses.

## Funding

These analyses were funded by AstraZeneca and Allergan. AstraZeneca's rights to ceftaroline fosamil were acquired by Pfizer in December 2016. Ceftaroline fosamil is being developed by Pfizer and AbbVie (following its acquisition of Allergan). All authors of this publication had access to the data required to develop the manuscript and take responsibility for the accuracy and integrity of these data.

## Data Sharing Statement

Upon request, and subject to certain criteria, conditions and exceptions (see https://www.pfizer.com/science/clinical-trials/trial-data-and-results for more information), Pfizer will provide access to individual deidentified participant data from Pfizer‐sponsored global interventional clinical studies conducted for medicines, vaccines and medical devices (1) for indications that have been approved in the United States and/or European Union or (2) in programs that have been terminated (ie, development for all indications has been discontinued). Pfizer will also consider requests for the protocol, data dictionary, and statistical analysis plan. Data may be requested from Pfizer trials 24 months after study completion. The deidentified participant data will be made available to researchers whose proposals meet the research criteria and other conditions, and for which an exception does not apply, via a secure portal. To gain access, data requestors must enter into a data access agreement with Pfizer.

## Supporting information

Supporting information

Supporting information

Supporting information

Supporting information

Supporting information
