# Supplementary material for: Pharmacokinetic and Pharmacodynamic Target Attainment in Adult and Pediatric Patients Following Administration of Ceftaroline Fosamil as a 5‐Minute Infusion
Source: Clin Pharmacol Drug Dev. 2021 Jan 19;10(4):420–7. doi: 10.1002/cpdd.907 (PMC8048922; doi:10.1002/cpdd.907)
Supplement: Supplementary file 1 — Supporting information [file CPDD-10-420-s001.docx]

## **Table S1**. Model-Predicted Median Steady-State Ceftaroline Exposure Parameters for Simulated Patients with Mild Renal Impairment (nCrCL ≥50 to <80 mL/min/1.73 m^2^) Receiving Ceftaroline Fosamil as 5-Minute and 60-Minute IV Infusions

| **Age Group** | **Dosage Regimen^a^** | **IV Infusion Duration** | **Weight (kg)^b^** | **C_max,ss_ (mg/L)^b^** | **C_max,ss_ Ratio^c^** | **AUC_ss,0–24_ (mg/L*h)^b^** | **AUC_ss,0–24_ Ratio^c^** | **%*f*T>  1 mg/L^b^** |
| --- | --- | --- | --- | --- | --- | --- | --- | --- |
| Adults | 600 mg q12h | 60 minutes | 77.6 (52.2, 105) | 25.1 (12.6, 48.9) | 1.26 | 127 (70, 235) | 0.98 | 78.5 (51.2, 100) |
|  |  | 5 minutes |  | 31.8 (14.4, 72.3) |  | 125 (69, 231) |  | 74.4 (47.1, 100) |
| >12 to <18 years | 12 mg/kg q8h | 60 minutes | 52.7 (36.7, 74.7) | 20.7 (11.5, 35.6) | 1.28 | 136 (81, 227) | 1.00 | 92.6 (65.4, 100) |
|  |  | 5 minutes |  | 26.5 (13.7, 51.2) |  | 136 (82, 226) |  | 87.7 (59.3, 100) |
| ≥6 to <12 years | 12 mg/kg q8h | 60 minutes | 28.6 (19.2, 46.8) | 28.8 (17.0, 45.4) | 1.32 | 175 (110, 276) | 1.00 | 93.8 (66.7, 100) |
|  |  | 5 minutes |  | 38.2 (20.5, 69.8) |  | 175 (109, 276) |  | 88.6 (60.5, 100) |
| ≥2 to <6 years | 12 mg/kg q8h | 60 minutes | 15.8 (11.8, 22.4) | 28.3 (17.5, 44.0) | 1.39 | 160 (102, 252) | 1.00 | 82.7 (59.3, 100) |
|  |  | 5 minutes |  | 39.3 (22.2, 69.6) |  | 160 (101, 249) |  | 77.8 (53.1, 100) |
| 18 to <24 months | 8 mg/kg q8h | 60 minutes | 11.7 (9.82, 14.1) | 20.6 (12.8, 32.3) | 1.34 | 133 (83, 219) | 1.00 | 88.9 (60.5, 100) |
|  |  | 5 minutes |  | 27.6 (15.5, 49.6) |  | 133 (83, 218) |  | 82.7 (54.3, 100) |
| 12 to <18 months | 8 mg/kg q8h | 60 minutes | 10.4 (8.58, 12.7) | 20.9 (13.0, 32.6) | 1.33 | 140 (86, 227) | 1.00 | 92.6 (63.0, 100) |
|  |  | 5 minutes |  | 27.7 (16.6, 49.1) |  | 140 (86, 228) |  | 87.7 (57.4, 100) |
| 6 to <12 months | 8 mg/kg q8h | 60 minutes | 8.43 (6.56, 10.8) | 21.4 (13.3, 33.3) | 1.30 | 152 (93, 247) | 1.00 | 97.5 (67.9, 100) |
|  |  | 5 minutes |  | 27.9 (16.0, 49.7) |  | 152 (93, 247) |  | 93.8 (61.7, 100) |
| 2 to <6 months | 8 mg/kg q8h | 60 minutes | 5.75 (4.11, 7.67) | 21.2 (13.2, 33.3) | 1.24 | 168 (103, 275) | 1.00 | 100 (77.8, 100) |
|  |  | 5 minutes |  | 26.6 (15.3, 46.5) |  | 168 (104, 275) |  | 98.8 (71.6, 100) |

%*f*T>MIC, percentage of time that free drug concentrations are above the minimum inhibitory concentration (MIC) of the bacteria during a dosing interval; AUC_ss,0–24_, area under the plasma concentration–time curve over 24 hours at steady-state; C_max,ss_, maximum plasma concentration for a dosing interval at steady-state; IV, intravenous; nCrCL, body surface area-normalized creatinine clearance; q8h, every 8 hours; q12h, every 12 hours.
^a^All q8h pediatric dosage regimens were up to a maximum of 400 mg based on weight.
^b^Values are median, 5^th^ and 95^th^ percentiles (corresponding to 90% prediction intervals) for pediatric subjects, and 2.5^th^ and 97.5^th^ percentiles (corresponding to 95% prediction intervals) for adults based on summary of 100 simulation trials.
^c^Ratios are for 5-minute to 60-minute IV infusions.
