# Supplementary material for: Pharmacokinetic and Pharmacodynamic Target Attainment in Adult and Pediatric Patients Following Administration of Ceftaroline Fosamil as a 5‐Minute Infusion
Source: Clin Pharmacol Drug Dev. 2021 Jan 19;10(4):420–7. doi: 10.1002/cpdd.907 (PMC8048922; doi:10.1002/cpdd.907)
Supplement: Supplementary file 2 — Supporting information [file CPDD-10-420-s004.docx]

## Table S2. Model-Predicted Median (90% Prediction Interval) Steady-State Ceftaroline Exposure Parameters for Simulated Patients with Moderate Renal Impairment (nCrCL ≥30 to <50 mL/min/1.73 m^2^) Receiving Ceftaroline Fosamil as 5-Minute and 60-Minute IV Infusions

| **Age Group** | **Dosage Regimen^a^** | **IV Infusion Duration** | **Weight (kg)^b^** | **C_max,ss_ (mg/L)^b^** | **C_max,ss_ Ratio^c^** | **AUC_ss,0–24_ (mg/L*h)^b^** | **AUC_ss,0–24_ Ratio^c^** | **%*f*T>  1 mg/L^b^** |
| --- | --- | --- | --- | --- | --- | --- | --- | --- |
| Adults | 400 mg q12h | 60 minutes | 77.6 (52.2, 105) | 18.9 (9.5, 36.7) | 1.22 | 114 (64, 210) | 0.98 | 88.4 (56.2, 100) |
|  |  | 5 minutes |  | 22.9 (10.6, 51.1) |  | 113 (63, 207) |  | 84.3 (52.9, 100) |
| >12 to <18 years | 8 mg/kg q8h | 60 minutes | 52.7 (36.7, 74.7) | 15.2 (8.57, 26.2) | 1.24 | 114 (68, 192) | 1.01 | 96.3 (67.9, 100) |
|  |  | 5 minutes |  | 18.8 (9.90, 35.9) |  | 115 (68, 191) |  | 92.6 (61.7, 100) |
| ≥6 to <12 years | 8 mg/kg q8h | 60 minutes | 28.6 (19.2, 46.8) | 21.2 (12.5, 33.4) | 1.27 | 147 (92, 232) | 1.00 | 97.5 (70.4, 100) |
|  |  | 5 minutes |  | 26.9 (14.9, 48.4) |  | 147 (91, 233) |  | 93.8 (64.2, 100) |
| ≥2 to <6 years | 8 mg/kg q8h | 60 minutes | 15.8 (11.8, 22.4) | 20.8 (12.9, 31.9) | 1.32 | 134 (85, 209) | 1.00 | 88.9 (61.7, 100) |
|  |  | 5 minutes |  | 27.4 (15.4, 48.3) |  | 134 (84, 209) |  | 84.0 (56.7, 100) |

%*f*T>MIC, percentage of time that free drug concentrations are above the minimum inhibitory concentration (MIC) of the bacteria during a dosing interval; AUC_ss,0–24_, area under the plasma concentration–time curve over 24 hours at steady-state; C_max,ss_, maximum plasma concentration for a dosing interval at steady-state; IV, intravenous; nCrCL, body surface area-normalized creatinine clearance; q8h, every 8 hours; q12h, every 12 hours.
^a^All q8h pediatric dosage regimens were up to a maximum of 266.7 mg based on weight.
^b^Values are median, 5^th^ and 95^th^ percentiles (corresponding to 90% prediction intervals) for pediatric subjects, and 2.5^th^ and 97.5^th^ percentiles (corresponding to 95% prediction intervals) for adults based on summary of 100 simulation trials.
^c^Ratios are for 5-minute to 60-minute IV infusions.
