# Supplementary material for: Pharmacokinetic and Pharmacodynamic Target Attainment in Adult and Pediatric Patients Following Administration of Ceftaroline Fosamil as a 5‐Minute Infusion
Source: Clin Pharmacol Drug Dev. 2021 Jan 19;10(4):420–7. doi: 10.1002/cpdd.907 (PMC8048922; doi:10.1002/cpdd.907)
Supplement: Supplementary file 3 — Supporting information [file CPDD-10-420-s005.docx]

## Table S3. Model-Predicted Median (90% Prediction Interval) Steady-State Ceftaroline Exposure Parameters for Simulated Patients with Severe Renal Impairment (nCrCL ≥15 to <30 mL/min/1.73 m^2^) Receiving Ceftaroline Fosamil as 5-Minute And 60-Minute IV Infusions

| **Age Group** | **Dosage Regimen^a^** | **IV Infusion Duration** | **Weight (kg)^b^** | **C_max,ss_ (mg/L)^b^** | **C_max,ss_ Ratio^c^** | **AUC_ss,0–24_ (mg/L*h)^b^** | **AUC_ss,0–24_ Ratio^c^** | **%*f*T>  1 mg/L^b^** |
| --- | --- | --- | --- | --- | --- | --- | --- | --- |
| Adults | 300 mg q12h | 60 minutes | 77.6 (52.2, 105) | 15.6 (8.0, 30.0) | 1.18 | 108 (62, 192) | 0.99 | 94.2 (62.8, 100) |
|  |  | 5 minutes |  | 18.4 (8.8, 40.4) |  | 106 (61, 190) |  | 91.7 (59.5, 100) |
| >12 to <18 years | 6 mg/kg q8h | 60 minutes | 52.7 (36.7, 74.7) | 13.4 (7.54, 23.1) | 1.17 | 120 (70, 212) | 1.01 | 100 (79.0, 100) |
|  |  | 5 minutes |  | 15.7 (8.48, 29.5) |  | 121 (70, 212) |  | 100 (74.1, 100) |
| ≥6 to <12 years | 6 mg/kg q8h | 60 minutes | 28.6 (19.2, 46.8) | 18.5 (11.0, 29.5) | 1.21 | 154 (93, 258) | 1.01 | 100 (84.0, 100) |
|  |  | 5 minutes |  | 22.4 (12.5, 39.4) |  | 156 (93, 257) |  | 100 (77.7, 100) |
| ≥2 to <6 years | 6 mg/kg q8h | 60 minutes | 15.8 (11.8, 22.4) | 17.9 (10.9, 28.2) | 1.26 | 141 (84, 233) | 1.01 | 98.8 (70.4, 100) |
|  |  | 5 minutes |  | 22.5 (12.9, 38.7) |  | 142 (83, 234) |  | 98.8 (65.4, 100) |

%*f*T>MIC, percentage of time that free drug concentrations are above the minimum inhibitory concentration (MIC) of the bacteria during a dosing interval; AUC_ss,0–24_, area under the plasma concentration–time curve over 24 hours at steady-state; C_max,ss_, maximum plasma concentration for a dosing interval at steady-state; IV, intravenous; nCrCL, body surface area-normalized creatinine clearance; q8h, every 8 hours; q12h, every 12 hours.
^a^All q8h pediatric dosage regimens were up to a maximum of 200 mg based on weight.
^b^Values are median, 5^th^ and 95^th^ percentiles (corresponding to 90% prediction intervals) for pediatric subjects, and 2.5^th^ and 97.5^th^ percentiles (corresponding to 95% prediction intervals) for adults based on summary of 100 simulation trials.
^c^Ratios are for 5-minute to 60-minute IV infusions.
