# Supplementary material for: Pharmacokinetic and Pharmacodynamic Target Attainment in Adult and Pediatric Patients Following Administration of Ceftaroline Fosamil as a 5‐Minute Infusion
Source: Clin Pharmacol Drug Dev. 2021 Jan 19;10(4):420–7. doi: 10.1002/cpdd.907 (PMC8048922; doi:10.1002/cpdd.907)
Supplement: Supplementary file 4 — Supporting information [file CPDD-10-420-s003.docx]

Table S4. Median PTA by Age for Ceftaroline Based on Simulations for Patients with Normal Renal Function or Renal Impairment Receiving Ceftaroline Fosamil as 5-Minute and 60-Minute IV Infusions

| **Age Group** | **Dosage Regimen^a^** | **% of Patients Achieving 35% *f*T>1 mg/L (*Staphylococcus aureus* PK/PD Target)** | | **% of Patients Achieving 44% *f*T>0.5 mg/L (*Streptococcus pneumoniae* PK/PD Target)** | | |
| --- | --- | --- | --- | --- | --- | --- |
|  |  | **60-Minute  IV Infusion** | **5-Minute  IV Infusion** | **60-Minute IV Infusion** | **5-Minute  IV Infusion** | |
| **Normal Renal Function (nCrCL ≥80 mL/min/1.73 m^2^)^a^** | | | | | |  |
| Adults | 600 mg q12h | 99.7 | 99.0 | 100 | 99.7 | |
| >12 to <18 years | 12 mg/kg q8h | 100 | 100 | 100 | 100 | |
| ≥6 to <12 years | 12 mg/kg q8h | 100 | 100 | 100 | 100 | |
| ≥2 to <6 years | 12 mg/kg q8h | 100 | 100 | 100 | 100 | |
| 18 to <24 months | 8 mg/kg q8h | 100 | 99.8 | 100 | 100 | |
| 12 to <18 months | 8 mg/kg q8h | 100 | 100 | 100 | 100 | |
| 6 to <12 months | 8 mg/kg q8h | 100 | 100 | 100 | 100 | |
| 2 to <6 months | 8 mg/kg q8h | 100 | 100 | 100 | 100 | |
| **Mild Renal Impairment** **(nCrCL ≥50 to <80 mL/min/1.73 m^2^)^a^** | | | | | |  |
| Adults | 600 mg q12h | 100 | 99.9 | 100 | 100 | |
| >12 to <18 years | 12 mg/kg q8h | 100 | 100 | 100 | 100 | |
| ≥6 to <12 years | 12 mg/kg q8h | 100 | 100 | 100 | 100 | |
| ≥2 to <6 years | 12 mg/kg q8h | 100 | 100 | 100 | 100 | |
| 18 to <24 months | 8 mg/kg q8h | 100 | 100 | 100 | 100 | |
| 12 to <18 months | 8 mg/kg q8h | 100 | 100 | 100 | 100 | |
| 6 to <12 months | 8 mg/kg q8h | 100 | 100 | 100 | 100 | |
| 2 to <6 months | 8 mg/kg q8h | 100 | 100 | 100 | 100 | |
| **Moderate Renal Impairment** **(nCrCL ≥30 to <50 mL/min/1.73 m^2^)^b^** | | | | | |  |
| Adults | 400 mg q12h | 100 | 100 | 100 | 100 | |
| >12 to <18 years | 8 mg/kg q8h | 100 | 100 | 100 | 100 | |
| ≥6 to <12 years | 8 mg/kg q8h | 100 | 100 | 100 | 100 | |
| ≥2 to <6 years | 8 mg/kg q8h | 100 | 100 | 100 | 100 | |
| **Severe Renal Impairment (nCrCL ≥15 to <30 mL/min/1.73 m^2^)^c^** | | | | | |  |
| Adults | 300 mg q12h | 100 | 100 | 100 | 100 | |
| >12 to <18 years | 6 mg/kg q8h | 100 | 100 | 100 | 100 | |
| ≥6 to <12 years | 6 mg/kg q8h | 100 | 100 | 100 | 100 | |
| ≥2 to <6 years | 6 mg/kg q8h | 100 | 100 | 100 | 100 | |

IV, intravenous; MIC, minimum inhibitory concentration; nCrCL, body surface area-normalized creatinine clearance; PTA, probability of target attainment; q8h, every 8 hours; q12h, every 12 hours.
Median values based on summary of 100 simulation trials.
^a^All q8h pediatric dosage regimens were up to a maximum of 400 mg based on weight.
^b^All q8h pediatric dosage regimens were up to a maximum of 266.7 mg based on weight.
^c^All q8h pediatric dosage regimens were up to a maximum of 200 mg based on weight.
