# Supplementary material for: Pharmacokinetic and Pharmacodynamic Target Attainment in Adult and Pediatric Patients Following Administration of Ceftaroline Fosamil as a 5‐Minute Infusion
Source: Clin Pharmacol Drug Dev. 2021 Jan 19;10(4):420–7. doi: 10.1002/cpdd.907 (PMC8048922; doi:10.1002/cpdd.907)
Supplement: Supplementary file 5 — Supporting information [file CPDD-10-420-s002.docx]

# Supplementary Methods

## Pediatric Patients and Adults with Normal Renal Function

### Population PK Model

A population pharmacokinetic (PK) dataset was developed by adding data from 5 pediatric studies to a pooled dataset from studies in healthy adult subjects and adult patients with complicated skin and soft-tissue infection (cSSTI) and community-acquired pneumonia (CAP).^1, 2^ Ceftaroline fosamil and ceftaroline population PK were described by a simultaneous modeling approach, with a 2-compartment disposition model for ceftaroline fosamil and a 2-compartment disposition model for ceftaroline. The most significant covariate on clearance (CL) was body surface area (BSA)-normalized creatinine CL (CrCL; calculated using the Cockcroft–Gault formula [mL/min] for adults and using the Schwartz bedside formula for pediatric patients [mL/min/1.73 m^2^]).^3, 4^ Other covariates evaluated as part of the full covariate model included age, weight, patient type (healthy subjects or patients with an infection) and BSA, as determined by the effect on ceftaroline CL, and the effect of weight and patient type on the central volume of distribution. In patients aged ≤2 years, an effect of renal function maturation as a function of postmenstrual age (PMA) on ceftaroline CL replaced the term representing the effect of BSA-normalized CrCL.

Population PK analyses were conducted using nonlinear mixed-effects modeling with a qualified installation of the nonlinear mixed-effects modeling (NONMEM) software, version 7.3 (ICON Development Solutions, Hanover, Maryland, USA). Assessment of model adequacy was driven by the data and guided by goodness-of-fit criteria, including (i) visual inspection of diagnostic scatter plots (observed vs. predicted concentration, residual/weighted residual/conditional, weighted residual vs. predicted concentration or time, and histograms of individual random effects); (ii) successful convergence of the minimization routine with at least 2 significant digits in parameter estimates; (iii) plausibility of parameter estimates; (iv) precision of parameter estimates; and (v) correlation between model parameter estimation errors <0.95.

### Monte Carlo Simulations

For adult simulations, covariates were generated from a multivariate normal distribution using covariate correlations observed in the population PK data in adult patients. Weights for pediatric age groups were based on US Centers for Disease Control Growth Charts.^5^ In pediatric patients aged ≤2 years, an effect of renal maturation function as a function of PMA on ceftaroline CL replaced the term representing the effect of BSA-normalized CrCL. Moreover, the renal maturation function (fractional change in clearance due to maturation, FPMA) was adjusted to allow for the simulation of renal impairment. FPMA was scaled from 0.625 (50/80) to 0.988 (79/80) for mild renal impairment, 0.375 (30/80) to 0.613 (49/80) for moderate renal impairment, and 0.125 (10/80) to 0.363 (29/80) for severe renal impairment. The divisor for the scaling was set to 80, as this is the lowest CrCL cut-off point for normal renal function. The dividend for the scaling was set to the upper and lower bounds of CrCL denoting mild (50/80–79/80), moderate (30/80–49/80), and severe (10/80–29/80) renal impairment.

Simulations were performed for the following ceftaroline fosamil dosage regimens: normal renal function/mild renal impairment (600 mg every 12 hours [q12h] for adults; 12 mg/kg [maximum 400 mg] every 8 hours [q8h] for those aged ≥2 to <18 years; 8 mg/kg [maximum 400 mg] q8h for those aged ≥2 to <24 months), moderate renal impairment (8 mg/kg [maximum 266.7 mg] q8h for those aged ≥2 to <18 years), and severe renal impairment (6 mg/kg [maximum 200 mg] q8h for those aged ≥2 to <18 years). The nCRCL categories for renal impairment were defined as Normal: ≥80 mL/min/1.73 m^2^; Mild: ≥50 to <80 mL/min/1.73 m^2^; Moderate: ≥30 to <50 mL/min/1.73 m^2^; Severe: <30 mL/min/1.73 m^2^. For normal renal function simulations, all subjects were assumed to have nCRCL of 150 mL/min/1.73 m^2^ (consistent with a flat relationship ≥80 mL/min/1.73 m^2^). For mild, moderate and severe renal impairment simulations for pediatric subjects, nCRCL was sampled from a uniform distribution with the interval set to the cutoff values for the specific renal function categories above.

In total, 100 simulations were performed for each dosage regimen and renal function category, with 600 (300 male and 300 female) patients in each 1-month age group from ≥2 months to <18 years (60,000 simulated patients total per age group/regimen). For adults with normal renal function, 300 patients were simulated for each dosage regimen for each of the 100 simulated data sets (30,000 simulated patients total per regimen).

## Adult Patients with Renal Impairment

### Population PK Model

A separate, previously unreported ceftaroline population PK model was used to simulate adult patients with mild, moderate or severe renal impairment. This model was based on a PK data set comprising 227 patients and 219 healthy subjects contributing 6241 measurable concentrations (1704 for ceftaroline fosamil and 4537 for ceftaroline). The data set was pooled from 16 phase 1–3 clinical studies (P903-01, P903-02, P903-03, P903-04, P903-06, P903-07, P903-08, P903-09, P903-11, P903-13, P903-14, P903-15, P903-17, P903-18, and P903-20; these studies were all included in the above adult and pediatric model^2^ and details are reviewed by Li et al 2019;^6^ and CPT-PK-05, a phase 1 study evaluating single doses of ceftaroline fosamil given by 5- and 60-minute IV infusions in healthy subjects). The model included adults with cSSTI or CAP, adolescent patients (aged ≥12–17 years) with an infection of any type requiring antibiotic therapy, and healthy adult subjects. Covariates included in the data set were age, weight, sex, patient status, BSA, Cockcroft–Gault-calculated CrCL based on total body weight in mL/min, and BSA-normalized CrCL. Derived covariates, such as BSA, CrCL, and BSA-normalized CrCL were calculated from the observed covariates. Population PK analyses were conducted using nonlinear mixed-effects modeling with a qualified installation of the NONMEM software, version 7.3 (ICON Development Solutions, Hanover, Maryland, USA). The first-order conditional estimation method was employed for all model runs.

The PK of ceftaroline fosamil and ceftaroline were described by a simultaneous modeling approach with 2-compartments for ceftaroline fosamil and 2-compartments for ceftaroline. The model was parameterized in terms of clearance of ceftaroline fosamil (CLcf), central volume of distribution of ceftaroline fosamil (Vccf), intercompartmental clearance for central and peripheral compartment 1 of ceftaroline fosamil (Q1cf), peripheral volume of distribution 1 of ceftaroline fosamil (Vp1cf), absorption rate constant 1 of ceftaroline fosamil (ka1cf), CLc, Vcc, intercompartmental clearance for central and peripheral compartment of ceftaroline (Qc), and peripheral volume of distribution of ceftaroline (Vpc). This model included effects of BSA-normalized CrCL on CLc for subjects with nCrCL <80 mL/min, a separate CL by dialysis for subjects undergoing dialysis, and effects of end-stage renal disease (ESRD) on CLc, age on CLc, and patient type on CLc and Vcc. Interindividual random effect distributions on CLcf, Vccf, ka1cf, CLc, Vcc, and Vpc were modeled as additive variance models, with a covariance term between CLcf, Vccf, CLc, and Vcc, while residual random effects were described with a combined additive and proportional model. A covariance term for the proportional residual random effects was implemented to account for the possible correlation between ceftaroline fosamil and ceftaroline concentrations within a sample, as both analytes were assayed from the same plasma sample. The adequacy of the final PK model and parameter estimates were investigated with a predictive check method.

### Monte Carlo Simulations

The final combined ceftaroline fosamil and ceftaroline population PK model from the previous population analysis^2^ was used to simulate 5- and 60-minute infusions for adult patients with mild, moderate or severe renal impairment. This was because (1) both models (current model with inclusion of CPT-PK-05 data versus previous model without CPT-PK-05) provided near identical model parameter estimates; (2) the fit for both models was adequate; (3) thus the nonparametric bootstrap for the model incorporating CPT-PK-05 data was not needed. Monte Carlo simulations for ceftaroline at steady state were performed using appropriate covariate distributions and a pre-defined level of parameter uncertainty. Covariates were generated from a multivariate normal distribution using covariate correlations observed in the population PK data in adult patients. Simulations were performed for various ceftaroline fosamil dosage regimens and renal impairment categories including mild renal impairment (600 mg q12h), moderate renal impairment (400 mg q12h), and severe renal impairment (300 mg q12h) at variable infusion durations; 300 patients were simulated for each dosage regimen and renal function category for each of 100 simulated data sets (30,000 simulated patients total per renal function group/regimen).

# References

1. Riccobene T, Jakate A, Rank D. A series of pharmacokinetic studies of ceftaroline fosamil in select populations: normal subjects, healthy elderly subjects, and subjects with renal impairment or end-stage renal disease requiring hemodialysis. *J Clin Pharmacol.* 2014;54(7):742-752.

2. Riccobene TA, Khariton T, Knebel W, et al. Population PK modeling and target attainment simulations to support dosing of ceftaroline fosamil in pediatric patients with acute bacterial skin and skin structure infections and community-acquired bacterial pneumonia. *J Clin Pharmacol.* 2017;57(3):345-355.

3. Cockcroft DW, Gault MH. Prediction of creatinine clearance from serum creatinine. *Nephron.* 1976;16(1):31-41.

4. Schwartz GJ, Munoz A, Schneider MF, et al. New equations to estimate GFR in children with CKD. *J Am Soc Nephrol.* 2009;20(3):629-637.

5. Centers for Disease Control. CDC Growth Charts. Available at: <http://www.cdc.gov/growthcharts/cdc_charts.htm>.

6. Li J, Das S, Zhou D, Al-Huniti N. Population Pharmacokinetic Modeling and Probability of Target Attainment Analyses in Asian Patients With Community-Acquired Pneumonia Treated With Ceftaroline Fosamil. *Clin Pharmacol Drug Dev.* 2019;8(5):682-694.
